# Proceedings of the 4th Annual United States Army Institute of Surgical Research Summer Undergraduate Research Internship Program 2016

**DOI:** 10.1186/s12967-017-1117-8

**Published:** 2017-02-22

**Authors:** Lauren E. Cornell, Whitney Greene, Rachel O. Haich, Lee C. Mangum, Gerardo R. Garcia, Charles H. Guymon, Kevin S. Akers, Jennifer Landry, Jennifer McDaniel, Gina Griffith, Elaine Por, David Rios, David Zamora, Brian Lund, Alexandra M. Forbes, Joel D. Newton, Timothy M. Mok, Josue Garcia-Marcano, Andres Penagos-Nino, Jeffrey D. Keesee, Xiaowu Wu, Harvey Harper, Bin Liu, Aaron M. Lewis, Martin G. Schwacha, Andrew P. Cap, Daniel N. Darlington, Allison E. Tempel, David Silliman, Rose Grimm, John Decker, Johanna Dungca, Remington Wong, Tyler Everett, Anders Carlsson, Rodney Chan, Kristen R. Lye, Alicia M. Schiller, Victor A. Convertino

**Affiliations:** 1 US Army Institute of Surgical Research, JBSA Fort Sam Houston, TX 78234 USA; 2 Auburn University, Auburn, AL 36849 USA; 3 Brooke Army Medical Center, JBSA Fort Sam Houston, TX 78234 USA; 4 University of Georgia, Athens, GA 40031 USA; 5 Washington State University, Spokane, WA 99204 USA; 6 North Carolina State University, Raleigh, NC 27695 USA; 7 Drexel University, Philadelphia, PA 19104 USA; 8 Florida Southern College, Lakeland, FL 33801 USA; 9 Dental and Craniofacial Trauma Research and Tissue Regeneration, US Army Institute of Surgical Research, JBSA Fort Sam Houston, TX 78234 USA; 10 Clinical Division and Burn Center, US Army Institute of Surgical Research, JBSA Fort Sam Houston, TX 78234 USA; 11 Quality Skin Collaborative for Advanced Reconstruction and Regeneration (Q-SCARR™), JBSA Fort Sam Houston, TX USA; 12 Texas Lutheran University, Seguin, TX 78155 USA

## I1 Proceedings of the 4th Annual Summer Undergraduate Research Internship Program 2016

### Lauren E. Cornell^1^, Whitney Greene^1^, Rachel O. Haich^2^, Lee C. Mangum^1^, Gerardo R. Garcia^1^, Charles H. Guymon^1^, Kevin S. Akers^1,3^, Jennifer Landry^4^, Jennifer McDaniel^1^, Gina Griffith^1^, Elaine Por^1^, David Rios^1^, David Zamora^1^, Brian Lund^1^, Alexandra M. Forbes^5^, Joel D. Newton^6^, Timothy M. Mok^7^, Josue Garcia-Marcano^1^, Andres Penagos-Nino^1^, Jeffrey D. Keesee^1^, Xiaowu Wu^1^, Harvey Harper, Jr.^1^, Bin Liu^1^, Aaron M. Lewis^1^, Martin G. Schwacha^1^, Andrew P. Cap^1^, Daniel N. Darlington^1^, Allison E. Tempel^1,8^, David Silliman^1^, Rose Grimm^1^, and John Decker^1^, Johanna Dungca^9^, Remington Wong^9^, Tyler Everett^9^, Anders Carlsson^9,11^, Rodney Chan^10^, Kristen R. Lye^1,12^, Alicia M. Schiller^1^ and Victor A. Convertino^1^

#### ^1^US Army Institute of Surgical Research, JBSA Fort Sam Houston, TX 78234, USA; ^2^Auburn University, Auburn, AL 36849, USA; ^3^Brooke Army Medical Center, JBSA Fort Sam Houston, TX 78234, USA; ^4^University of Georgia, Athens, GA 40031, USA; ^5^Washington State University, Spokane, WA 99204, USA; ^6^North Carolina State University, Raleigh, NC 27695, USA; ^7^Drexel University, Philadelphia, PA 19104; USA; ^8^Florida Southern College, Lakeland, FL 33801, USA; ^9^Dental and Craniofacial Trauma Research and Tissue Regeneration, US Army Institute of Surgical Research, JBSA Fort Sam Houston, TX 78234, USA; ^10^Clinical Division and Burn Center, US Army Institute of Surgical Research, JBSA Fort Sam Houston, TX 78234, USA; ^11^Quality Skin Collaborative for Advanced Reconstruction and Regeneration (Q-SCARR™), JBSA Fort Sam Houston, TX, USA; ^12^Texas Lutheran University, Seguin, TX 78155, USA

*Journal of Translational Medicine* 2017, **15 (Suppl 2)**: I1

**About the summer undergraduate intern program:** The research mission of the US Army Institute of Surgical Research (USAISR) is to improve the quality of life and care of injured service members. Each summer, the USAISR invites a select number of undergraduate students from accredited colleges and universities to participate in a student intern program. This program is designed to allow students who are interested in careers in engineering, science, or medicine to participate in ongoing research efforts at the USAISR. Upon acceptance, the interns work closely with a USAISR staff mentor in one of the following research task areas: resuscitation, hemorrhage control, burn injury, blood and coagulation, ocular trauma, dental trauma, tactical combat casualty care research, intensive care, pain, regenerative medicine, and extremity trauma.

The mentored research experience exposes undergraduate research interns to the latest developments in integrated technology, science and engineering solutions for health care at the nation’s premier military trauma research institute.

Interns are given an overview of the task areas within the USAISR, and are briefed on health, safety, and security policies. To further enhance their experience at the USAISR students are given the opportunity to observe medical rounds in the Burn ICU, and attend weekly journal club meetings, where they are required to present at least one session. The 2016 summer internship program ran from 06 June to 10 August 2016.

**Eligibility:** To be eligible for the USAISR summer undergraduate research internship program, students must have completed at least their first year of study in an accredited Bachelor’s degree program. Majors focused on engineering or sciences are preferred. Additionally, all prospective students must hold U.S. citizenship to apply.

**Meeting format:** After 10 weeks of research in their respective departments, interns present their findings to the research community at the USAISR. They must prepare a poster following institution guidelines and present to the USAISR and San Antonio Military Medical Center (SAMMC) scientific community, which allows for critical interaction with clinicians and scientists. Attendees include military personnel, clinicians, post doctoral fellows, staff scientists, and principal investigators (PIs).

**Awards:** Upon successful completion of the summer internship and poster presentations, interns were awarded with a certificate of appreciation from Acting Director of Research Kevin Chung, for their diligence and contributions to improving the quality of care for wounded service men and women.

**Conclusions:** The summer internship program is mutually beneficial to both interns and USAISR researchers. Interns are exposed to real life laboratory experiences such as experimental design, troubleshooting, optimizing protocols, data analysis, and scientific reporting. Interns are also exposed to the realities of combat-related injuries sustained by members of the United States military. These experiences culminate in a formal presentation of their work to established investigators from both clinical and research specialties.

Researchers at the USAISR benefit from the energy, enthusiasm, and novel perspectives the interns bring to their projects, which may lead to new discoveries and research directions.

**Acknowledgements:** We would like to acknowledge the USAISR Research Directorate for their commitment to the continuation of this summer internship program and the support of this publication effort. Specifically we would like to thank, the former Acting Director of Research Kevin Chung, the current Director of Research Anthony Pusateri, Deputy Director of Research Kevin Akers, and Ms. Susan Walker. We would also like to acknowledge the summer intern program coordinator, David Burmeister, and the individual departments and PIs for committing time and effort to mentor these interns. The USAISR has contracted with the Oak Ridge Institute for Science and Education for stipend support of student participants.

**Disclaimers:** The opinions and assertions contained herein are the private views of the authors and are not to be construed as official or reflecting the views of the Department of Defense or Departments of the Army. This research debuted below was funded by the U.S. Army Medical Research and Materiel Command. These studies were conducted under protocols reviewed and approved by the US Army Medical Research and Materiel Command Institutional Review Board and in accordance with the approved protocols.

**Sponsorship:** Publication charges for this supplement were funded by the USAISR.

**Fig. 1:**
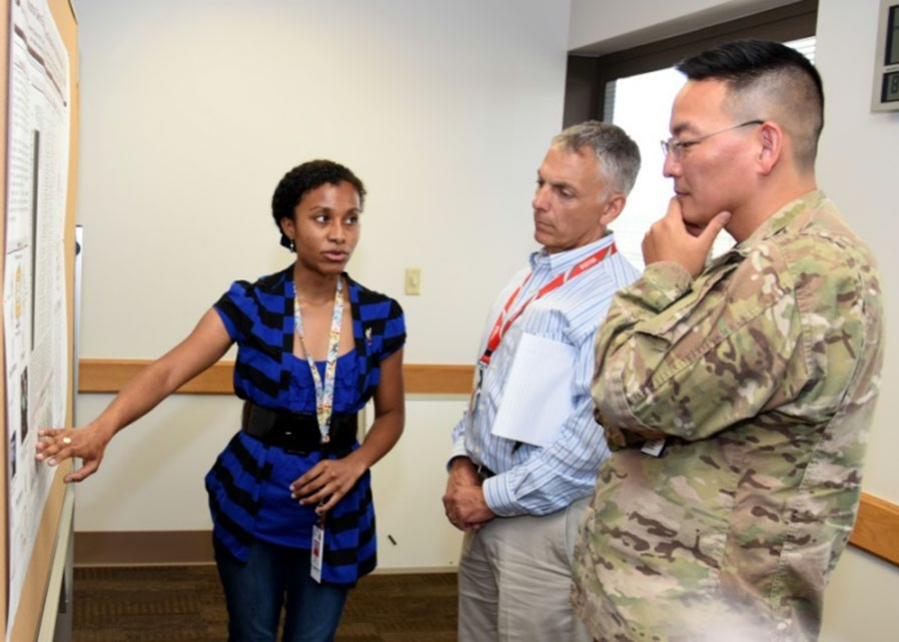
Summer intern presenting her work to Anthony Pusateri (*left:*) and Kevin Chung (*right:*). Photo credit: U.S. Army Photo by Dr. Steven Galvan, US Army Institute of Surgical Research Public Affairs

**Consent:** The authors have written informed consent from the people in Fig. 1 to publish the image.

**Application website:** http://usaisr.amedd.army.mil/2016_undergrad_internship.html (accessible from the USA.)

Here is an alternative source of information about the internship, which can be accessed outside of the USA: https://orise.orau.gov/media-center/news-releases/2016/army-institute-surgical-research-summer-internships-accepting-applications.aspx.

## P1 A review of the USAISR clinical isolate collection

### Rachel O. Haich^1^, Lee C. Mangum^2^, Gerardo R. Garcia^2^, Charles H. Guymon^2^ and Kevin S. Akers^2,3^

#### ^1^Auburn University, Auburn, AL 36849, USA; ^2^US Army Institute of Surgical Research, JBSA Fort Sam Houston, TX 78234, USA; ^3^Brooke Army Medical Center, JBSA Fort Sam Houston, TX 78234, USA

##### **Correspondence**: Kevin S. Akers

*Journal of Translational Medicine* 2017, **15 (Suppl 2)**: P1

**Background:** The clinical isolate collection housed within the US Army Institute of Surgical Research (USAISR) consists of approximately 71,000 bacterial isolates obtained from patients in the USAISR Burn Unit over a span of nearly 40 years. Thus far, there has been no systematic evaluation of the effectiveness of the storage and organization methods used for this collection. The objectives of this study were to determine the viability of wound and blood isolates of *Klebsiella pneumoniae, Acinetobacter baumannii:*, and *Pseudomonas aeruginosa:* within the repository and to evaluate changes in biofilm production over time in representative isolates.

**Materials and methods:** Information for each isolate is currently stored in hardbound log books in addition to an electronic Oracle database. The repository database was queried to find wound and blood isolates identified as *K. pneumoniae, A. baumannii:*, or *P. aeruginosa:* and these entries were cross-refere*:*nced with the physical log books to identify storage location numbers. Isolates that could be physically located in the repository were streaked for growth on TSA with 5% sheep blood. Biofilm formation capacity was evaluated in successfully cultured isolates using a standard crystal violet staining-based in vitro assay [1].

**Results:** 1037 isolates from the database met our selection criteria for inclusion in this experiment. Of these, 343 isolates (33.1%) could be physically located and retrieved. 150 of these isolates (43.7% of retrievable) were found to be viable. The overall successful recovery rate was 14.5% with individual species recovery rates of 13.1% for *K. pneumoniae:*, 23.4% for *A. baumannii:*, and 11.0% for *P. aeruginosa:*. No *K. pneumoniae:* isolates stored before 1997 were found to be viable. *A. baumannii:* and *P. aeruginosa:* were recoverable in all time periods tested. Significant variation in biofilm production was observed among isolates of the same species, with positive biofilm formation occurring in 35.7% of *K. pneumoniae:* isolates, 18.0% of *A. baumannii:* isolates, and 47.7% of *P. aeruginosa:* isolates.

**Conclusions:** Given the relatively small proportion of isolates successfully located, cultured, and evaluated in this study (14.5% of total attempted), a review of storage methods and sample organization could be beneficial to ensure the continued utility of the collection. Routine subculture may be necessary in order to preserve the isolates over the long term and reduce the unnecessary storage of nonviable specimens. Although variation in biofilm production was observed within each species based on collection date, no distinct trends were evident over the study period for any species tested.

**Reference**

1. Stepanovic’ S, Vukovic D, Dakic I, Savic B, Svabic-Vlahovic M. A modified microtiter-plate test for quantification of staphylococcal biofilm formation. J Microbiol Methods. 2000;40(2):175–9.

## P2 The effects of low-level blast in vivo

### Jennifer Landry^1^, Lauren Cornell^2^, Jennifer McDaniel^2^, Gina Griffith^2^, Elaine Por^2^, David Rios^2^, Brian Lund^2^ and David Zamora^2^

#### ^1^University of Georgia, Athens, GA 40031, USA; ^2^US Army Institute of Surgical Research, JBSA Fort Sam Houston, TX 78234, USA

##### **Correspondence**: David Zamora

*Journal of Translational Medicine* 2017, **15 (Suppl 2)**: P2

**Background:** Ocular trauma has become the 4th most prevalent combat injury among recent military conflicts in Iraq and Afghanistan [1]. The typical soldier requiring hospitalization is between ages 20 and 24 and may have decreased quality of life and lose the ability to return to combat [2]. Improvised explosive devices (IEDs) have significantly increased the prevalence of blast injuries incurred by soldiers, making understanding the effects of blasts imperative. This survey of studies aimed to provide the student the opportunity to participate in various stages of the research process. As such, a number of different studies are referenced. The main objectives of these studies were to mimic blast conditions for soldiers on the battlefield in order to find better therapeutics and to test the effectiveness of potential therapeutics in the “wet lab.”

**Materials and methods:** Blast model 1: adult male rats (275–300 g) were anesthetized and delivered a low level blast (68.0 ± 2.7 kPa) created by a compressed air shock tube. In order to assess the effects of repeat and single blasts on pain and inflammation signaling pathways, low level blasts were delivered either once or were repeated daily for five consecutive days.

Blast model 2: adult male, Dutch belted rabbits (1.9 ± 0.03 kg) were anesthetized and placed into the expansion cone of the compressed-air-driven shock tube facing into the direction of the incident blast wave. Animals were subjected to a BOP of 42.5–164.1 kPa (~22 psi) with a positive phase specific impulse of 199.6–228.5 kPa-ms. Rabbits were allowed to recover for 48 h, and then euthanized and ocular tissues collected for immunohistochemical, biochemical and molecular analyses.

**Results:** Animal model 1(Rat): immunostaining showed significant infiltration of neutrophils into the cornea after repeated blasts as compared to control animals. Additionally, increases in TRPV1, CGRP, SP and ET-1, proteins and peptides involved in pain transmission, were observed in the cornea following single and repeated blast exposure.

Animal model 2 (Rabbit): immunohistological staining showed an increase in AQP-4 expression in the retina after blast. There was also an increase in GFAP expression in the optic nerve.

**Conclusions:** Animal model 1 (Rat): up-regulation of TRPV1, CGRP, SP and ET-1 as well as neutrophil infiltration provides a basis for further studies to investigate low level blast and its effect on ocular physiology and function.

Animal model 2 (Rabbit): increase in both GFAP and AQP-4 indicate that there may be a link between blast exposure and edema.

**References**

1. Gundogan FC, Akay F, Yolcu, U, Uzun S, Ilhan A, Toyran S, Eyi E, Diner O. Ocular blast injuries related to explosive military ammunition. J R Med Corps. 2016;162(1):39–43.
2. Hilber DJ. Eye injuries, active component, U.S. Armed Forces, 2000–2010. MSMR. 2011;18(5):2–7.

## P3 Cyclic nucleotides in platelets influence platelet aggregation

### Alexandra M. Forbes^1^, Joel D. Newton^2^, Timothy M. Mok^3^, Josue Garcia-Marcano^4^, Andres Penagos-Nino^4^, Jeffrey D. Keesee^4^, Xiaowu Wu^4^, Andrew P. Cap^4^ and Daniel N. Darlington^4^

#### ^1^Washington State University, Spokane, WA 99204, USA; ^2^North Carolina State University, Raleigh, NC 27695, USA; ^3^Drexel University, Philadelphia, PA 19104, USA; ^4^US Army Institute of Surgical Research, JBSA Fort Sam Houston, TX 78234, USA

##### **Correspondence**: Daniel N. Darlington

*Journal of Translational Medicine* 2017, **15 (Suppl 2)**: P3

**Background:** Platelets aggregation makes up 70–80% of clot strength and deficits in platelet function may strongly influence the hemostatic ability of trauma patients. Severe polytrauma is associated with coagulopathy in approximately 33% of trauma patients. It has been shown that platelet function falls after trauma in patients [1]. We have recently shown that platelets aggregation is compromised after trauma in rat. One hypothesis that has been proposed is that intracellular levels of cAMP and cGMP are elevated after trauma, and elevated cAMP and cGMP inhibits platelet aggregation, including aggregation to normal agonist, like adenosine diphosphate (ADP) and collagen. The objective of this study was to determine if elevation of cAMP or cGMP effects platelet aggregation to ADP and collagen [2–5].

**Materials and methods:** Human whole blood was collected in citrate tubes (4 ml) from healthy donors in accordance with an institutional standard operating procedure. Tubes were centrifuged without at 200 g for 10 min and the platelet rich plasma (PRP) was removed. 1 ml of PRP was incubated at various times with either PGI_2_ (prostaglandin I_2_) or SNAP (S-Nitroso-*N:*-acetylpenicillamine) to stimulate adenylyl and guanylyl cyclase respectively. Platelet aggregation was measured by impedance aggregometry (Multiplate, Roche) and induced using either ADP (1 mM) or collagen (1 mg/ml). Cyclic Nucleotides were extracted from 0.5 ml of PRP with 2.25 ml EtOH, 0.1% formic acid and 10 mM cGMP-Br, separated on a c18 column (Phenomenex) using a water/MeCN 0.1% formic acid gradient, and measured on a TSQ Quantiva LS-MS/MS (ThermoFischer).

**Results:** Normal human platelets were stimulated with either PGI_2_ or SNAP showed a significant elevation in cAMP and cGMP respectively. This elevation was significant for 3 h. Both ADP and collagen stimulated platelets aggregation. This stimulation was inhibited by PGI_2_. SNAP partially inhibited the ADP-induced platelet aggregation, but had no effect on collagen-induced aggregation.

**Conclusions:** The inhibition of platelet aggregation is correlated with rise in both cAMP and cGMP. These two cellular intermediates may act to control platelet function in the resting and activated state.

**References**

1. Cap AP, Hunt B. Acute Traumatic Coagulopathy. Curr Opin Crit Care. 2014;20:638–45.
2. Smolenski A. Novel roles of cAMP/cGMP-dependent signaling in platelets. J Thromb Haemost. 2012;10(2):167–76.
3. Rivera J, Luisa M, Navarro-Nunez L, Vicente V. Platelet receptors and signaling in the dynamic of thrombus formation. Haematologica. 2009;94(5):700–11.
4. Li Z, Delaney K, O’Brien KA, Du X. Signaling during platelet adhesion and activation. Arterioscler Thromb Vasc Boil. 2010;30(12):2341–9.
5. Savage B, Cattaneo M, Ruggeri ZM. Mechanisms of platelet aggregation. Curr Opin Hematol. 2001;8(5):270–6.

## P4 Cyclic nucleotides are influenced by classic agonists in platelets

### Joel D. Newton^1^, Alexandra M. Forbes^2^, Timothy M. Mok^3^, Andres Penagos-Nino^4^, Josue Garcia-Marcano^4^, Xiaowu Wu^4^, Andrew P. Cap^4^ and Daniel N. Darlington^4^

#### ^1^North Carolina State University, Raleigh, NC 27695, USA; ^2^Washington State University, Spokane, WA 99204, USA; ^3^Drexel University, Philadelphia, PA 19104, USA; ^4^US Army Institute of Surgical Research, JBSA Fort Sam Houston, TX 78234, USA

##### **Correspondence**: Daniel N. Darlington

*Journal of Translational Medicine* 2017, **15 (Suppl 2)**: P4

**Background:** Platelet aggregation makes up to 70–80% of clot strength and deficits in platelet function may strongly influence the hemostatic ability of trauma patients. Severe polytrauma is associated with coagulopathy in approximately 33% of trauma patients and this coagulopathy may be due, in part, to platelet dysfunction. It has been shown that platelet dysfunction occurs in trauma patients, and a recent rat model has shown that platelets ability to aggregate is compromised after trauma [1]. It has been proposed that intracellular levels of cAMP and cGMP are elevated after trauma. Elevated cAMP and cGMP inhibits platelet aggregation, including aggregation to normal agonist [2–5], like adenosine diphosphate (ADP), thrombin (PAR1), epinephrine, collagen and thromboxane (U46619, TXA agonist). The objective of this study was to determine if these classic platelet agonists change the intracellular levels of cAMP or cGMP.

**Materials and methods:** Human whole blood was collected in citrate tubes in accordance with institutional standard operating procedures. Whole blood was centrifuged at 200 g for 10 min to generate platelet rich plasma (PRP). 1 ml of PRP was incubated at various times with either PGI_2_ (prostaglandin I_2_) or SNAP (S-Nitroso-*N:*-acetylpenicillamine) to stimulate adenylyl and guanylyl cyclase respectively. Platelet aggregation was measured by impedance aggregometry induced using either ADP (1 mM) or collagen (1 mg/ml). Cyclic nucleotides were then extracted from 0.5 ml of PRP with 2.25 ml EtOH, 0.1% NH4 formate and 10 mM cGMP-Br as an internal standard. Cyclic nucleotides were separated on a c18 column using a water/MeCN 0.1% formic acid gradient, and measured on a TSQ Quantiva LS-MS/MS. Cyclic nucleotides concentration was divided by the platelet count for normalization of samples.

**Results:** ADP, PAR1, collagen, EPI and U46619 were found to have no effect on resting levels of either cAMP or cAMP. However, stimulation of adenylyl or guanylyl cyclase by PGI2 or SNAP elevated cAMP or cGMP respectively. The addition of ADP, PAR1, and EPI was found to prevent an elevation of cAMP induced by PGI2. Collagen and U46619 had no effect on the cAMP levels. All 5 agonists were found to inhibit the rise in cGMP induced by SNAP.

**Conclusion:** Platelet activation by ADP, thrombin, collagen, TXA or EPI does not involve changes in cyclic nucleotides when the platelet is in the resting state. However, when cyclic nucleotides levels are augmented, ADP, thrombin and EPI will inhibit generation of cAMP, and all classic agonist will inhibit generation of cGMP.

**References**

1. Cap AP, Hunt B. Acute Traumatic Coagulopathy. Curr Opin Crit Care. 2014;20:638–45.
2. Rivera J, Luisa M, Navarro-Nunez L, Vicente V. Platelet receptors and signaling in the dynamic of thrombus formation. Haematologica. 2009;94(5):700–11.
3. Li Z, Delaney K, O’Brien KA, Du X. Signaling during platelet adhesion and activation. Arterioscler Thromb Vasc Boil. 2010;30(12):2341–9.
4. Savage B, Cattaneo M, Ruggeri ZM. Mechanisms of platelet aggregation. Curr Opin Hematol. 2001;8(5):270–6.
5. Smolenski A. Novel roles of cAMP/cGMP-dependent signaling in platelets. J Thromb Haemost. 2012;10(2):167–76.

## P5 TLR-2 stimulation leads to platelet aggregation and potentiates ADP and collagen

### Timothy M. Mok^1^, Joel D. Newton^2^, Alexandra M. Forbes^3^, Harvey Harper, Jr.^4^, Bin Liu^4^, Aaron M. Lewis^4^, Xiaowu Wu^4^, Martin G. Schwacha^4^, Andrew P. Cap^4^ and Daniel N Darlington^4^

#### ^1^Drexel University, Philadelphia, PA 19104, USA; ^2^North Carolina State University, Raleigh, NC 27695, USA; ^3^Washington State University, Spokane, WA 99204, USA; ^4^US Army Institute of Surgical Research, JBSA Fort Sam Houston, TX 78234, USA

##### **Correspondence**: Daniel N. Darlington

*Journal of Translational Medicine* 2017, **15 (Suppl 2)**: P5

**Background:** Severe trauma can lead to cellular damage and release of intracellular substances that are not normally seen outside the cell or in the plasma. These substances can vary in molecular size and type (proteins, peptides, nucleic acids) and are ligands for various pattern recognition receptors, including Toll-like Receptors (TLR) [1]. These ligands have been collectively termed damage associated molecular patterns (DAMPs) [2]. TLR-2, -4 and 9 have been found in many cell types, including platelets [3]. Because platelet aggregation is affected by severe trauma, we set out to determine if stimulation of platelets with agonist to TLR-2, -4 and -9 affects the ability of platelets to aggregate. The objective of this study was to determine if agonists to TLR-2, -4, and -9 will cause platelet aggregation, and affect aggregation by classic platelet activators ADP and collagen.

**Materials and methods:** Whole blood was collected over PPACK from normal human volunteers and platelet rich plasma (PRP) generated by gentle centrifugation (200 g for 20 min), and washed once in Tyrodes buffer to remove plasma. PGI2 10 nM was added to prevent activation during wash. Platelets were diluted in Tyrodes buffer to a platelet count of 250,000/ml. Platelet aggregation was measured in a 96 well plate containing 170 μl of PRP, 20 ml of agonist. Agonist used to stimulate aggregation included ADP (1 mM) and collagen (1 mg/ml). Aggregation was measured by an increase in turbidity by absorbance at l = 595 nm before and 20 min after the addition of agonist. Standard aggregation curves were generated by varying the concentration of ADP and collagen. The effects of PAM3 (1 mg/ml, TLR-2 agonist), LPS (5 × 106 EU, TLR-4 agonist), and dsDNA (200 mM, TLR-9 agonist) were determined on platelet aggregation with and without varying dose of ADP or collagen.

**Results:** Platelet aggregation was stimulated by ADP, collagen and by TLR-2, but not TLR-4 or -9 agonists.

Aggregation cause by TLR-2 agonist suggested that there may have been potentiation of the ADP and Collagen. ADP or collagen given at various concentrations showed a dose-dependent rise in platelet aggregation. Co-stimulation withTLR-2 agonist move the ADP and collagen dose response curve up and to the left.

**Conclusions:** Stimulation of TLR-2 receptors on platelets leads to aggregation AND potentiates the effect of ADP and collagen. This suggests that DAMPS may act through TLR-2 to potentiate aggregation during trauma. This potentiation may be good at the site of trauma, where strong clots are necessary, but may be detrimental if aggregation occurs away from the site of trauma, potentially causing thrombosis.

**References**

1. Zhang Q, Raoof M, Chen Y, Sumi Y, Sursal T, Junger W, Brohi K, Itagaki K, Hauser CJ. Circulating mitochondrial DAMPs cause inflammatory responses to injury. Nature. 2010;464(7285):104–7.

2. Matzinger P. The Danger Model: A Renewed Sense of Self. Science. 2002;296(5566):301–5.

3. Cognasse F, Hamzeh H, Chavarin P, Acquart S, Genin C, Garraud O. Evidence of Toll-like receptor molecules on human platelets. Immunol Cell Biol. 2005;83(2):196–8.

## P6 Efficacy of decalcification methodologies on rat calvarial caps

### Allison E. Tempel^1,2^, David Silliman^2^, Rose Grimm^2^, and John Decker^2^

#### ^1^Florida Southern College, Lakeland, FL 33801, USA; ^2^US Army Institute of Surgical Research, San Antonio, TX 78234, USA

##### **Correspondence**: John Decker

*Journal of Translational Medicine* 2017, **15 (Suppl 2)**: P6

**Background:** During recent U.S. military operations, head and neck trauma accounted for one-third of sustained injuries with mandibular fractures having the highest prevalence [1]. Mandibular insufficiency and bone inadequacy for dental implant therapy is a common outcome following recovery. Ongoing rat calvarial studies evaluate regenerative bone products using non-decalcified histology. Processing non-decalcified histology is expensive, time-consuming, and specialized. Previous studies using variable formic acid (FA) decalcification and hematoxylin and eosin (H&E) staining exhibited minimal tissue loss and improved histological images [2]. Our objective is to identify a possible surrogate for non-decalcified histology. This study assessed the effects of formalin and FA duration and concentration on rat calvarial histology.

**Materials and methods:** Ninety rat calvarial caps were divided in half and fixated in formalin for either one or two months. Samples were evenly distributed into groups and decalcified in 5, 10, or 20% FA solution for 7, 14, or 21 days. After decalcification all samples were sectioned, embedded in paraffin, and stained with H&E. Each histology slide was then evaluated and scored using a qualitative histological pathology scale and the average numerical value per group was analyzed [3].

**Results:** Decreased formalin fixation time was associated with improved histological images. Five percent FA at 21 days yielded the highest average optimal histological score and 20% FA at 21 days was associated with extremely poor histological scores.

**Conclusions:** Further investigation is required but initially findings suggest that a shorter fixation period followed by decalcification in a low FA solution over a longer time period provides optimal bone histology for histometric analysis.

**References**

1. Lew TA, Walker JA, Wenke JC, Blackbourne LH, Hale RG. Characterization of Craniomaxillofacial Battle Injuries Sustained by United States Service Members in the Current Conflicts of Iraq and Afghanistan. J Oral Maxillofac Surg. 2010;68(1):3–7.
2. Sanjai K, Kumarswamy J, Patil A, Papaiah L, Jayaram S, Krishnan L. (2012). Evaluation and comparison of decalcification agents on the human teeth. J Oral Maxillofac Pathol. 2012;16(2):222–7.
3. Brown RW. Histologic Preparations: Common Problems and Their Solutions. 1st ed. Northfield, Ill: College of American Pathologists; 2009.

## P7 Transient analysis of α-SMA in porcine partial thickness burns

### Johanna Dungca^1^, Remington Wong^1^, Tyler Everett^1^, Anders Carlsson^1,3^ and Rodney Chan^2^

#### ^1^Dental and Craniofacial Trauma Research and Tissue Regeneration, US Army Institute of Surgical Research, JBSA Fort Sam Houston, TX 78234, USA; ^2^Clinical Division and Burn Center, US Army Institute of Surgical Research, JBSA Fort Sam Houston, TX 78234, USA; ^3^Quality Skin Collaborative for Advanced Reconstruction and Regeneration (Q-SCARR™), JBSA Fort Sam Houston, TX, USA

##### **Correspondence**: Rodney Chan

*Journal of Translational Medicine* 2017, **15 (Suppl 2)**: P7

**Background:** Burn injuries account for up to 20% of military casualties in combat warfare. Scar contractions are commonly seen in burn injuries [1]. Scar contraction is a result of dysregulated and prolonged proliferation of myofibroblasts expressing alpha smooth muscle actin (α-SMA) [2]. The α-SMA protein is normally expressed in both smooth muscle and blood vessels, however normal fibroblast do not express the protein. When fibroblasts differentiate into myofibroblasts, expression of α-SMA is upregulated, enabling the contractile phenotype. Previous studies have shown an association of α-SMA expression with depth of burn, time to re-epithelialization, scar contraction and thickness [3]. This study compared transient scar contraction with α-SMA expression levels in a porcine partial-thickness burn model.

**Materials and methods:** This study was conducted in compliance with the Animal Welfare Act, the implementing Animal Welfare Regulations, and the principles of the Guide for the Care and Use of Laboratory Animals. Thermal contact burns were induced in a red Duroc porcine model and scar formation was followed for 120 days. Initial injury depth was established on biopsies from day 7 using a histological injury score. The histologic score, ranging from intact epithelium (0) to epithelial death in hair bulbs and glands (4), was used to grade the injury depth. Wound and scar assessments with photos and tissue biopsies was conducted at days 7, 14, 28, 60, 90 and 120 post-burn injury. Two biopsies were harvested during each assessment. One biopsy was used for histology and the other snap-frozen in liquid nitrogen and used for protein analysis. These frozen biopsies were used to analyze α-SMA expression using enzyme-linked immunosorbent assays (ELISAs).

**Results:** Quantitative photo analysis of wounded areas showed significant contraction compared to growth control, with a peak contraction at day 60 to 90 for all injury scores. The preliminary data suggest that increased α-SMA expression levels correlate with peak scar contraction. Scars from deep-partial thickness injuries expressed significantly higher levels of α-SMA than normal controls on day 60 (Score 3 p > 0.01, Score 4 p > 0.05). A significant difference was also observed between the most shallow injuries and partial-thickness injuries (Score 2 vs. Score 3 p > 0.01).

**Conclusions:** The preliminary results from this study suggest that increased α-SMA expression correlates with peak scar contraction in this model of porcine scarring. Furthermore, burn depth was found to be a predictor of α-SMA expression and subsequent wound contraction.

**References**

1. Atiyeh BS, Gunn SW, Hayek SN. Military and civilian burn injuries during armed conflicts. Ann Burns Fire Disasters. 2007;20(4):203–15.
2. Bochaton-Piallat ML, Gabbiani G, Hinz B. The myofibroblast in wound healing and fibrosis: answered and unanswered questions. F1000Research. 2016;5:752.
3. Wang XQ, Kravchuk O, Winterford C, Kimble RM. The correlation of in vivo burn scar contraction with the level of α-smooth muscle actin expression. Burns. 2011;37(8):1367–77.

## P8 Effectiveness of R-to-R interval variability analysis in distinguishing mechanisms of autonomic control of heart rate during exercise and hemorrhage

### Kristen R. Lye^1,2^, Alicia M. Schiller^2^ and Victor A. Convertino^2^

#### ^1^Texas Lutheran University, Seguin, TX 78155, USA; ^2^US Army Institute of Surgical Research, JBSA Fort Sam Houston, TX 78234, USA

##### **Correspondence**: Victor A. Convertino

*Journal of Translational Medicine* 2017, **15 (Suppl 2)**: P8

**Background:** Exercise and hemorrhage are two conditions that elicit similar compensatory mechanisms to protect adequate tissue oxygenation [1, 2]. One such compensatory mechanism shared by exercise and hemorrhage is the increase in heart rate that acts to maintain cardiac output and tissue perfusion pressure (i.e., arterial blood pressure).The autonomic nervous system (ANS), which controls heart rate, is comprised of the sympathetic (SNS) and parasympathetic (PNS) nervous systems. Measurement of high (HF) and low (LF) frequency power spectra have been used as a non-invasive technique to study the contributions of the PNS and SNS to control heart rate. By measuring the variability (frequencies) of R-to-R intervals (RRI), differences in SNS and PNS activity can be estimated and used to compare ANS activity during exercise or hemorrhage. We hypothesized that that the contributions of the SNS and PNS to the elevation in heart rate would be similar during exercise and simulated hemorrhage.

**Materials and methods:** Eleven human subjects underwent progressive reductions in central blood volume similar to those induced by blood loss by using lower body negative pressure (LBNP) as a model of hemorrhage. Stages of LBNP were determined by progressive 5-min levels of chamber decompression at -15, -30, -45, and -60 mmHg. No less than 11 days later, the same individuals underwent an exercise protocol in which each subject performed workloads on a recumbent bike that were determined by individually matching the heart rate achieved during the last 3 min of the parallel LBNP level. HF and LF were then derived from the RRI and set as a ratio (i.e., HF/LF and LF/HF) to normalize the autonomic balance between the PNS and SNS.

**Results:** During increased intensity of exercise or LBNP, a progressive reduction in HF/LF (PNS activity) was statistically indistinguishable between exercise (180 ± 60 to 75 ± 50) and LBNP (215 ± 100 to 45 ± 10) while a progressive increase in LF/HF (SNS activity) was statistically indistinguishable between exercise (175 ± 12 to 425 ± 300) and LBNP (180 ± 15 to 800 ± 405).

**Conclusion:** The results from this experiment suggest that the magnitude of PNS (vagal) withdrawal and SNS activation contribute similarly as mechanisms underlying the elevation in heart rate during the early compensatory phase of progressive exercise and hemorrhage.

**References**

1. Rickards CA, Ryan KL, Cooke WH, Romero SA, Convertino VA. Combat stress or hemorrhage? Evidence for a decision-assist algorithm for remote triage. Aviat Space Environ Med. 2008;79: 670–6.
2. Ryan KL, Rickards CA, Hinojosa-Laborde C, Gerhardt RT, Cain J, Convertino VA. Advanced technology development for remote triage applications in bleeding combat casualties. US Army Med Dep J. 2011: 61–72.

